# Are phosphodiesterase Type 5 inhibitors potential therapies for Alzheimer’s disease and related dementias?

**DOI:** 10.1093/braincomms/fcac260

**Published:** 2022-10-11

**Authors:** Danielle Newby

**Affiliations:** Nuffield Department of Orthopaedics, Rheumatology and Musculoskeletal Sciences, Centre for Statistics in Medicine, University of Oxford, Oxford, UK

## Abstract

This scientific commentary refers to ‘No association between initiation of phosphodiesterase-5 inhibitors and risk of incident Alzheimer’s disease and related dementia: results from the Drug Repurposing for Effective Alzheimer’s Medicines (DREAM) study’ by Desai et al. (https://doi.org/10.1093/braincomms/fcac247)

**This scientific commentary refers to ‘No association between initiation of phosphodiesterase-5 inhibitors and risk of incident Alzheimer’s disease and related dementia: results from the Drug Repurposing for Effective Alzheimer’s Medicines (DREAM) study’ by Desai *et al.* (https://doi.org/10.1093/braincomms/fcac247) in *Brain Communications.***

Drug treatments to prevent or treat dementia are urgently needed. Existing drug treatments such as acetylcholinesterase inhibitors do not stop the disease from getting worse and only treat symptoms temporarily for a subset of patients. As the pathology underlying dementia begins decades before symptoms appear, and has numerous modifiable risk factors, this provides a window of opportunity to intervene through repurposing existing drugs known to be effective for these mechanisms.^1^ Existing medications approved to treat other health conditions have been shown to modify some dementia-related risk factors such as high blood pressure and diabetes. Repurposing is a highly cost-effective strategy for developing new treatments, as it does not involve the cost and safety concerns associated with trialling a new compound.^2^ However, the repurposing of drugs for dementia is not without its critics. There are concerns about the validity of results due to the discordance between observational research studies and importantly discordance with randomized control trials. Much of this can be put down to limitations in observational data such as heterogeneity of populations and failure to address biases (e.g. reverse causation and confounding) as well as a lack of triangulation from different types of methods, studies and data.

In this issue of *Brain Communications*, Desai and colleagues^3^ undertook an extensive analytical approach to investigate if phosphodiesterase type 5 (PDE-5) inhibitors (sildenafil and tadalafil) could be potential therapies for Alzheimer’s disease and related dementias (ADRD). The study triangulated evidence from medical records, comparing PDE-5 users versus endothelin receptor antagonists’ users in patients with pulmonary arterial hypertension, as well as testing the impact of sildenafil on cell culture based phenotypic assays for a variety of endophenotypes related to Alzheimer’s disease such as tau phosphorylation and amyloid-β. The study showed that PDE-5 inhibitors were not associated with ADRD risk in observational analysis and sildenafil did not ameliorate abnormalities relevant to Alzheimer’s disease in most cell culture–based phenotypic assays although did appear to have slight anti-inflammatory effects.

Phosphodiesterase type 5 inhibitors are marketed for the treatment of erectile dysfunction and arterial pulmonary arterial hypertension due to their vasodilatory properties. Evidence from cell and animal studies suggest PDE-5 inhibitors have potential benefits for neuroprotection.^4^ One study has indicated that sildenafil is associated with a 69% reduction in Alzheimer’s disease risk and showed using induced pluripotent stem cells from patients with Alzheimer’s disease, sildenafil increased neurite growth and decreased phospho-tau expression.^5^ A recent clinical study has shown tadalafil does not increase cerebral blood flow in older people with symptomatic small vessel disease, the main cause of vascular cognitive impairment.^6^

The study by Desai *et al.*^3^ contradicts the study by Fang *et al*.^5^ by showing no beneficial effects of PDE-5 inhibitors with ADRD risk. However, there are differences in how the study was conducted, what biases were addressed and therefore how the results can be interpreted. The main issue with pharmacoepidemiological studies is making clear the population of patients you are studying and what groups of patients you are comparing as this is important in how you can interpret the results. Desai *et al.*^3^ focused on a population of patients with pulmonary arterial hypertension and compared users of PDE-5 inhibitors with users of endothelin receptor antagonists, whereas Fang *et al.*^5^ compared users of sildenafil versus nonusers as well as comparing with users taking medications for cardiometabolic disease. Differences in study designs and failure to address or minimize biases can lead to incorrect conclusions and poor-quality evidence for regulatory and clinical practice. Therefore, it is important to address or minimize biases with suitable study designs and carry out additional analysis that can test the robustness of findings as shown by Desai *et al.*^3^ who carried out four main analyses (as well as subgroup analyses) to test for different biases such as informative censoring, reverse causality and outcome misclassification. Furthermore, carrying out analyses in multiple databases from different countries and different healthcare systems could also be a practical approach to increase confidence in results and potentially detect any biases.

Other biases such as residual confounding can be tested using negative control outcome analysis which could also be implemented.^7^ Negative controls are known conditions, procedures, medications that have no relationship between PDE-5 inhibitors and ADRD. If negative controls have an association with ADRD this implies there is residual confounding which might affect the results and interpretation. In addition to these biases, confounding by indication is one the main sources of bias in comparative effectiveness studies and its importance is not well appreciated. It is where the clinical indication or severity of disease influences treatment and is an independent risk factor for the study outcome. One method to minimize confounding by indication is to restrict the study population in a study to a single indication.^8^ For Desai *et al*.,^3^ the authors restricted the study population to a single indication of patients with pulmonary arterial hypertension. The lack of restriction to a single indication by Fang *et al.*^5^ potentially explains the large beneficial effects of sildenafil in this study. One issue with restriction for rarer diseases such as pulmonary arterial hypertension is the reduction in sample size and generalizability, as patients with pulmonary arterial hypertension have higher cardiometabolic comorbidities and may not be representative of individuals at risk for ADRD as pointed out by the current study. Further considerations adopted by Desai *et al.*^3^ are the importance of transparency and reproducibility with study design and analyses. The publication or registration detailing the study design and statistical analysis plan should be common practice before the study is carried out to ensure transparency and reduce publication bias.

The use of cell culture–based phenotypic assays can help understand potential mechanistic insights regarding the impact of how a drug works to ameliorate Alzheimer’s disease and related phenotypes such as amyloid-β, neuroinflammation and tau phosphorylation. Careful consideration of the dosages used for these assays so they reflect dosages that are clinically meaningful will be crucial. It is important to note that currently, most cell culture–based assays cannot precisely replicate human pathophysiology but despite this have vastly increased our understanding. Newer cell-based models are being developed that consider multiple cell types and other biological processes which will help increase understanding of how potential drug candidates work against Alzheimer’s disease endophenotypes and related pathologies.^9^

The clinical implications of this work and its context are important to recognize. This work showed in patients with pulmonary arterial hypertension, users of PDE-5 inhibitors compared to user’s endothelin receptor antagonists do not have a difference in ADRD risk as well as cell-based assays not showing amelioration of certain Alzheimer’s disease pathologies. It is difficult using observational data to determine if PDE-5 inhibitors versus nonusers decrease dementia risk due to the risk of confounding by indication and other biases. However, with the recent explosion in the availability of large-scale genetic association data, methods such as Mendelian randomization could allow the elucidation of causal relationships between different diseases and drug treatments and will be important (if plausible) for triangulation of evidence to strengthen these findings.^10^

The work of Desai *et al.*^3^ presents an important approach for evaluating the potential benefits of candidate drugs already on the market in dementia research. It carries out robust analyses lacking in many other studies as well as using cell-based assays to triangulate evidence and support their results. The triangulation of evidence by utilizing both real world data and laboratory work helps to provide solid grounds as basis for reliable conclusions. Furthermore, the current work poses further questions for future research including the impact of blood–brain barrier penetrating ability, the potential use of genetics and highlights the need for other researchers to adopt these transparent and robust analyses for drug repurposing studies for dementia.

## Data Availability

Data sharing is not applicable to this article as no new data were created or analysed.

## References

[1] Livingston G , Huntley J, Sommerlad A, et al Dementia prevention, intervention, and care: 2020 report of the lancet commission. Lancet. 2020;396(10248):413–446. 32738937 10.1016/S0140-6736(20)30367-6 PMC7392084

[2] Pushpakom S , Iorio F, Eyers PA, et al Drug repurposing: Progress, challenges and recommendations. Nat Rev Drug Discov. 2019;18(1):41–58. 30310233 10.1038/nrd.2018.168

[3] Desai RJ , Mahesri M, Lee SB, et al No association between initiation of phosphodiesterase-5 inhibitors and risk of incident Alzheimer’s disease and related dementia: Results from the Drug Repurposing for Effective Alzheimer’s Medicines (DREAM) study. Brain Commun. 2022. Published online October 4. 10.1093/braincomms/fcac247 PMC9598543 36330433

[4] Zuccarello E , Acquarone E, Calcagno E, et al Development of novel phosphodiesterase 5 inhibitors for the therapy of Alzheimer’s disease. Biochem Pharmacol. 2020;176. 10.1016/j.bcp.2020.113818 PMC7263960 31978378

[5] Fang J , Zhang P, Zhou Y, et al Endophenotype-based in silico network medicine discovery combined with insurance record data mining identifies sildenafil as a candidate drug for Alzheimer’s disease. Nature Aging. 2021;1(12):1175–1188. 35572351 10.1038/s43587-021-00138-z PMC9097949

[6] Pauls MMH , Binnie LR, Benjamin P, et al The PASTIS trial: Testing tadalafil for possible use in vascular cognitive impairment. Alzheimers Dement. 2022. Published online 2022. 10.1002/alz.12559 PMC10078742 35135037

[7] Lipsitch M , Tchetgen Tchetgen E, Cohen T. Negative controls: A tool for detecting confounding and bias in observational studies. Epidemiology. 2010;21(3):383. 20335814 10.1097/EDE.0b013e3181d61eeb PMC3053408

[8] Psaty BM , Siscovick DS. Minimizing bias due to confounding by indication in comparative effectiveness research: The importance of restriction. JAMA. 2010;304(8):897–898. 20736474 10.1001/jama.2010.1205

[9] Slanzi A , Iannoto G, Rossi B, Zenaro E, Constantin G. In vitro models of neurodegenerative diseases. Front Cell Dev Biol. 2020;8:328. 32528949 10.3389/fcell.2020.00328 PMC7247860

[10] Gill D , Georgakis MK, Walker VM, et al Mendelian randomization for studying the effects of perturbing drug targets. Wellcome Open Res. 2021;6:16. 33644404 10.12688/wellcomeopenres.16544.1 PMC7903200

